# Splenic rupture as the presenting manifestation of primary splenic angiosarcoma in a teenage woman: a case report

**DOI:** 10.1186/1752-1947-2-133

**Published:** 2008-04-29

**Authors:** Andreas Manouras, Panagiotis Giannopoulos, Levon Toufektzian, Haridimos Markogiannakis, Emmanuel E Lagoudianakis, Artemisia Papadima, Dimitrios Papanikolaou, Konstantinos Filis, Panagiotis Kekis

**Affiliations:** 1 1st Department of Propaedeutic Surgery, Hippokrateion Hospital, Athens Medical School, University of Athens, Q. Sofias 114 avenue, 11527 Athens, Greece; 2 Department of Anesthesiology, Hippokrateion Hospital, Q. Sofias 114 avenue, 11527 Athens, Greece

## Abstract

**Introduction:**

Primary splenic angiosarcoma is a rare neoplasm of vascular origin carrying a very poor prognosis, partly due to its high metastatic potential. This disease presents frequently with splenic rupture and hemorrhage. We report the case of a 17-year-old woman who presented with rupture of a primary splenic angiosarcoma.

**Case presentation:**

The patient presented with diffuse abdominal pain and distention. Clinical examination revealed severe tenderness in the left upper abdominal quadrant, a palpable abdominal mass, and hemodynamic instability with a systolic arterial blood pressure of 75 mmHg and heart rate of 135 beats per minute. Blood tests revealed anemia (hemoglobin 7.0 g/dl) and thrombocytopenia (platelets 70 × 10^9^/liter). After initial fluid resuscitation and stabilization, abdominal ultrasound and computed tomography were performed, revealing a large quantity of intraperitoneal free fluid, an enlarged spleen, and a heterogeneous low-density signal within the splenic parenchyma, which showed varying degrees of contrast enhancement. At laparotomy a huge (weight 1530 g, diameter 19 cm) actively bleeding spleen was identified and splenectomy was performed. Histopathology showed a primary splenic angiosarcoma. After an uneventful recovery, the patient was discharged on the sixth postoperative day.

**Conclusion:**

Primary splenic angiosarcoma is rare. Although this malignancy is usually encountered in advanced age, there have been a few reported cases among younger patients. The case reported here presented with splenic rupture, was treated by laparotomy and splenectomy, and the patient is disease free 16 months after surgery.

## Introduction

Primary angiosarcoma or hemangiosarcoma of the spleen is an extremely rare malignancy, the pathogenesis of which is not completely understood, with high metastatic potential and an exceedingly poor prognosis, regardless of the treatment regimen. This aggressive disease entity usually presents in adults in their sixth to seventh decade with only eight cases reported in the literature in people aged below 18 years of age. The reported median survival rates range from 4.4 months to 14 months, depending respectively on whether the diagnosis is made after splenic rupture or is based on clinical findings [1,2].

These mesenchymal tumors can easily be overlooked and splenic rupture is the most frequently encountered presenting manifestation. We report a case of splenic rupture due to angiosarcoma in a 17-year-old woman. This is the ninth reported case of this condition in a patient aged under 18 years of age and, to the best of the authors' knowledge, is the second case presenting with splenic rupture in this age group.

## Case presentation

**Figure 1:**
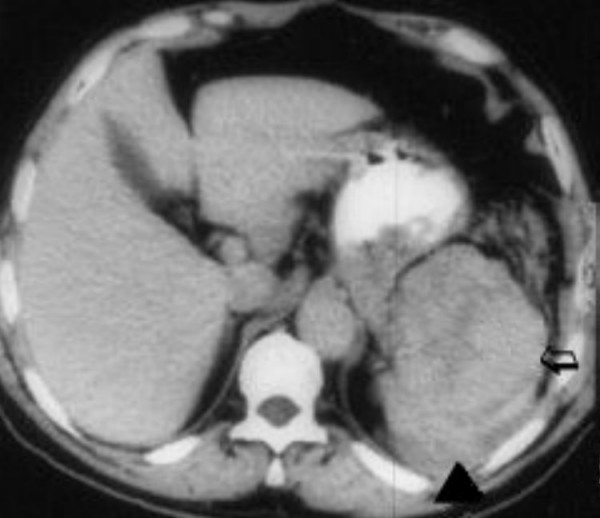
Abdominal computed tomography scan demonstrating intraperitoneal free fluid (arrowhead) and an enlarged, anechoic spleen with hypoechoic areas representing focal necrosis (arrow).

A 17-year-old woman presented to the emergency department of our hospital with diffuse abdominal pain and distention. Physical examination revealed severe tenderness in the left upper abdominal quadrant, a palpable abdominal mass, and hemodynamic instability; in particular, the patient's systolic arterial blood pressure was 75 mmHg and her heart rate was 135 beats per minute. After initial resuscitation with the administration of isotonic fluids, the hemodynamic status of the patient was normalized and further investigations were performed. Apart from moderate weight loss and sporadic episodes of dizziness, the reported medical history was unremarkable up to the day before admission. Laboratory investigations revealed anemia (hemoglobin 7.0 g/dl) and thrombocytopenia (platelets 70 × 10^9^/liter). An abdominal ultrasound showed a large quantity of intraperitoneal free fluid and an enlarged spleen. These findings were confirmed by an abdominal computed tomography (CT) scan (Figure 1), which further demonstrated hemorrhage originating from the spleen and a heterogeneous, low-density signal within the splenic parenchyma, which showed varying degrees of contrast enhancement. Chest X-ray and CT chest scan were normal.

Laparotomy revealed a huge, actively bleeding spleen; more than one liter of blood had filled the intraperitoneal cavity. No abnormal adhesions were detected between the spleen, the diaphragm, or other organs, and macroscopic examination of all four abdominal quadrants did not reveal any other pathology. Splenectomy was performed along with peritoneal lavage with warm saline to evacuate the clots. Intraoperatively, the patient received two units of packed red blood cells, one unit of platelets and two units of fresh frozen plasma.

**Figure 2:**
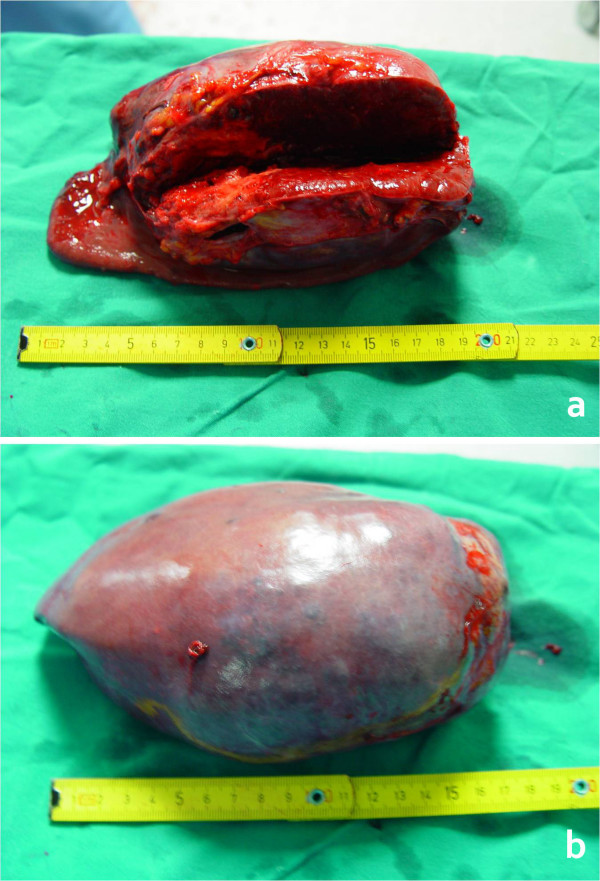
A – Excision specimen of the enlarged spleen. B – Hemorrhagic and nodular lesions excluded the diagnosis of idiopathic rupture and prompted further abdominal exploration for other pathologies.

**Figure 3:**
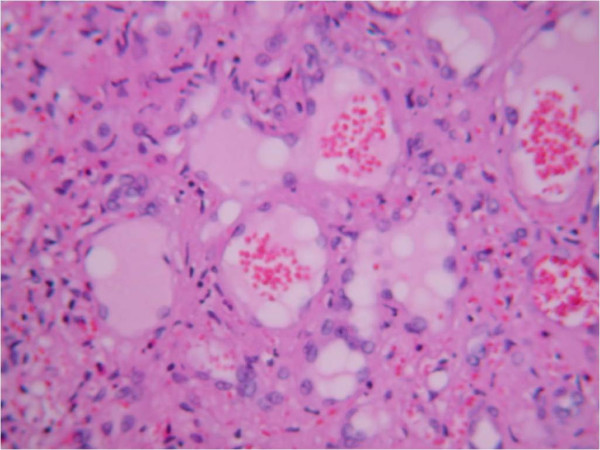
Histopathological findings of angiosarcoma of the spleen. Spindle-cell neoplasm has replaced the normal red and white pulp in the spleen whereas ectatic vascular spaces lined with hypertrophied endothelial cells are apparent. Hematoxylin and eosin stain ×80.

The splenic weight was 1530 g, its greatest diameter was 19 cm, and macroscopically it appeared nodular and spongy with hemorrhagic characteristics (Figure 2A and 2B), excluding the diagnosis of idiopathic rupture. The pathology examination of the excised spleen demonstrated an angiosarcoma, presumably of splenic origin (Figure 3). On immunohistochemical examination, the resected specimen showed positive immunostaining for CD31 and CD34. Owing to the highly metastatic potential of this malignant entity, and in addition to the negative macroscopic abdominal examination, a further CT scan of the abdomen, pelvis, and chest was performed with no evidence of metastatic foci.

After an uneventful recovery, the patient was discharged on the sixth postoperative day. The patient remains symptom and disease free after a follow-up period of 16 months, in contrast to the median survival of 4.4 months of patients with primary angiosarcoma of the spleen and associated splenic rupture [2].

## Discussion

Angiosarcoma originating from the spleen is a very rare mesenchymal malignant tumor of vascular origin, consisting of atypical and anaplastic endothelial cell concentrations. Other primary foci include the skin, soft tissues, breast and bone marrow. Its main histological feature is vascular channel formation with a sarcomatous stroma and papillary appearance due to endothelial cell proliferation, although an undifferentiated neoplasm is also a typical finding. Angiosarcoma is the most common of the primary malignant splenic tumors, which affect 0.14 to 0.25 per million people. This neoplasm shows a slight male predominance, with no predilection for race, geographic location or inheritability [3]. Overall, since 1879 when Langhans described the first case of angiosarcoma of the spleen, there have been approximately 200 cases reported in the literature. Splenic angiosarcoma is a disease of adulthood, with a mean age of presentation at 50 to 60 years among the various series, although the pediatric population may be also affected at virtually any age [4,5].

Although the natural history of this tumor is not clear, ionizing radiation, arsenic, vinyl chloride and chemotherapy for lymphoma have been implicated as causative factors, and it has been reported that the pathogenesis of splenic angiosarcoma requires the existence of a benign pathology such as a hemangioma or hemangioendothelioma. Recently, a case of angiosarcoma of the splenic capsule was reported and correlated with inert foreign body tumorigenesis [6]. There was no evidence of any of these factors in our case. This tumor has a high incidence of early metastasis as reflected in the reported rates of between 69% and 100% among various series, with the most common sites being the liver, lungs, bones or bone marrow, lymph nodes, gastrointestinal tract, brain and adrenal glands. Furthermore, synchronous malignancies of the breast, colon, skin and kidney have been reported. Our patient, however, did not show any evidence of either metastatic or synchronous disease and this is in accordance with the fact that younger patients usually present with a single focus of malignancy.

The clinical manifestations of primary splenic angiosarcoma range from asymptomatic disease to splenic rupture and lethal hemorrhage. Non-specific symptoms such as left upper abdominal discomfort, dizziness and weight loss, as well as signs such as thrombocytopenia and an enlarged spleen, which is present in more than of 50% of cases, are not diagnostic and may be easily associated with other pathologic conditions. Anemia, present in 70% of cases, may be of normocytic, schistocytic or megaloblastic type. Splenic rupture occurs in 13% to 32% of patients, and is the presenting and most serious manifestation in the majority of cases. It is the worst prognostic factor for survival, because it puts patients at increased risk of peritoneal dissemination with direct implantation of neoplastic tissue or vascular access and hematogenous spread. This finding is verified by the fact that splenectomy before rupture of the organ is accompanied by better survival rates [2]. Other independent prognostic factors include mitotic counts, tumor size and mode of treatment [7] as well as the thick capsule of the tumor [8].

Splenic rupture represents a serious complication, frequently encountered in patients with angiosarcoma of the spleen, and results in a fatal outcome in a significant proportion of cases. The presence of consumption coagulopathy with profound fibrinogenopenia and thrombocytopenia may lead to the development of disseminated intravascular coagulation, characterized by a lack of systemic fibrinolysis activation and profuse intraperitoneal hemorrhage, further decreasing survival rates [9].

Imaging modalities are invaluable for the differential diagnosis between other benign and malignant splenic tumors, although diagnostic accuracy is lacking. CT scans may reveal an enlarged spleen with hypo- or hyperattenuating areas on nonenhanced scans. Areas of hyperattenuation are likely to reflect acute hemorrhage or hemosiderin deposits. Contrast enhancement may be similar to that of a hepatic cavernous hemangioma, although the pattern of enhancement is variable. On contrast-enhanced scans, there may be peripheral or heterogeneous contrast enhancement. On MRI, ill-defined nodular lesions with low- or high-signal intensity may be seen on both T1- and T2-weighted images depending on the age of blood products and the presence of necrosis. High-signal intensity is related to subacute hemorrhage or tumor necrosis and low-signal intensity is related to chronic hemorrhage or fibrosis in the tumor. Massive splenic calcifications on CT scan or MRI have been reported as a prominent suggestive finding of angiosarcoma [10]; however, calcifications may also be found in patients with benign lesions such as a hemangioma [11].

## Conclusion

Splenic angiosarcoma, although rare, must be considered in the differential diagnosis of patients with hematologic abnormalities of unexplained origin and parenchymal lesions on spleen imaging. Definitive diagnosis requires laparotomy and splenectomy, since the risk of splenic rupture is enhanced with percutaneous splenic biopsies. Since metastatic disease is encountered in the majority of cases in patients with splenic angiosarcoma, the surgical approach with splenectomy is a more diagnostic than therapeutic modality. Although no effective chemotherapeutic protocol for angiosarcoma has yet been established, combined treatment with cyclophosphamide, adriamycin, vincristine and prednisone or other agents after splenectomy has been employed in a few cases, with fewer relatively good results [12]. As there was no evidence of metastatic disease, our patient was not treated with adjuvant chemotherapy; excellent results at 16 months of follow-up support this decision.

## Competing interests

The authors declare that they have no competing interests.

## Authors' contributions

AM carried out the operation and contributed to acquisition of consent and critical review of the manuscript. PG contributed to manuscript conception, research, acquisition of data, drafting and writing of the manuscript. LT contributed to manuscript conception, research, acquisition of data, drafting and writing of the manuscript. HM contributed to manuscript conception, research, acquisition of data, drafting and writing of the manuscript. EEL contributed to manuscript conception, research, acquisition of data, drafting and writing of the manuscript. AP contributed to organizing, drafting and critical review of the manuscript. DP assisted in the operation and contributed to writing of the manuscript. KF contributed to organizing and drafting of the manuscript, and critically revised the manuscript. PK assisted in the operation and contributed to critical review of the manuscript. All authors read and approved the final manuscript.

## Consent

Written informed consent was obtained from the patient for publication of this case report and any accompanying images. A copy of the written consent is available for review by the Editor-in-Chief of this journal.
